# Supplementary material for: An ex vivo model of interactions between extracellular vesicles and peripheral mononuclear blood cells in whole blood
Source: J Extracell Vesicles. 2023 Dec 4;12(12):12368. doi: 10.1002/jev2.12368 (PMC10694845; doi:10.1002/jev2.12368)
Supplement: Supplementary file 1 — Supporting Information [file JEV2-12-12368-s001.docx]

***Supplementary Materials***

***Figure S1.*** **Isolation and characterization of EVs produced by PalmGRET-transfected Expi293F cells.** (A) Schematic summarizing the workflow used to isolate EVs from large-scale cultures of Expi293F cells transfected with pLenti-PalmGRET dual reporter. Created with BioRender.com. (B-E) NFCM analysis of MemGlow-labeled and unlabeled PalmGRET EVs. (B,C) MemGlow-labeled PalmGRET EVs were diluted 50-fold in PBS and (D,E) unlabeled PalmGRET EVs were diluted 10,000-fold for analysis by NFCM. Histograms represent the particle size distribution and concentration of MemGlow-labeled (B) and unlabeled PalmGRET EVs (D). (C,E) Bivariate dot-lot plots of GFP fluorescence versus SS-A for EV samples analyzed by NFCM.

***Figure S2.*** **Relationship between particle concentration and nanoluciferase signal intensity.** Two-fold serial dilutions of MemGlow-labeled PalmGRET-EVs in DPBS starting from a concentration of 2.0E+10 EV/ml were performed 22X to determine the limit of detection of PalmGRET-EVs in nanoluciferase assays. (A) A strong linear relationship between PalmGRET-EV concentration (EV/mL) and nanoluciferase signal (in terms of relative light units, RLU) was observed for the first 16 dilution samples (R^2^=0.9939). (B) EVs at a concentration lower than 1.64E+06 EV/mL exhibited a weaker correlation of particle concentration and nanoluciferase signal (R^2^=0.6842).

***Figure S3.* Representative flow cytometry plots of PBMCs showing the gating scheme to identify immune cell subtypes.** Cells were identified by side scattering (SSC) and forward scatter (FSC) patterns. PBMC subtypes were identified using an antibody panel: monocytes (CD159^-^ CD3^-^ CD20^-^ and CD14^+^ or CD14^-^), T cells (CD3^+^ and CD4^+^ or CD8^+^), B cells (CD3^-^ CD20^+^), and NK cells (CD159^+^).

***Figure S4.* Representative flow cytometry plots of PalmGRET (EGFP)- and MemGlow-labeled EVs detected in association with PBMC subtypes after 24 h.** FITC (GFP) and Alexa Fluor 700 (MemGlow) overlay flow cytometry dot plots and histogram plots of the major PBMC subpopulations from whole blood incubated with PBS (red data points & histograms) or MemGlow-PalmGRET EVs (blue data points & histogram) over 24 hours. EVs were detected based on GFP and MemGlow signal. Plots are representative of n = 2, whole-blood collected from 2 different pig-tailed macaques on the same day.

***Figure S5.* Characterization of U-87 MG-derived EVs.** Schematic illustration of EV separation from U-87 conditioned cell culture media using ultracentrifugation (box 1) or by SEC (box 2). Figure was created using biorender.com. Nano flow cytometry measurement of particle size distribution and particle concentration of UC-purified U-87 EVs (B) and SEC-purified U-87 EVs (C). Western blot analysis of U-87 whole cell lysates (WCL), UC-purified U-87 EVs, and SEC-purified U-87 EVs (D). Size distribution and particle concentration profile of UC-purified U-87-EVs (E) and SEC-purified U-87-EVs (F) after MemGlow labeling.

***Figure S6.* PalmGRET-EVs labeled with MemGlow interact with PBMCs similarly to unlabeled PalmGRET EVs.** Cell-associated GFP signal was quantified by flow cytometry after 30 min of whole blood exposure to 8E+08 EVs labeled with (MG GFP EVs) or without MemGlow (GFP EVs). Flow cytometry bar graphs represent the mean of n = 4, whole-blood collected from 4 independent pig-tailed macaque donors and error bars represent the standard error of the mean. Statistical comparisons for GFP+ CD20+ B cells and GFP+ monocytes from whole blood exposed to GFP EVs or MG GFP EVs were performed using two-way ANOVA with Tukey’s post-hoc test, ns = not significant.
